# The Effects of Chunghyul-Dan, an Agent of Korean Medicine, on a Mouse Model of Traumatic Brain Injury

**DOI:** 10.1155/2017/7326107

**Published:** 2017-06-08

**Authors:** Won-Woo Choi, Kyungjin Lee, Beom-Joon Lee, Seong-Uk Park, Jung-Mi Park, Chang-Nam Ko, Youngmin Bu

**Affiliations:** ^1^Department of Clinical Korean Medicine, Graduate School, Kyung Hee University, Seoul 02447, Republic of Korea; ^2^College of Korean Medicine, Kyung Hee University, Seoul 02447, Republic of Korea

## Abstract

Chunghyul-Dan (CHD) is the first choice agent for the prevention and treatment of stroke at the Kyung Hee Medical Hospital. To date, CHD has been reported to have beneficial effects on brain disease in animals and humans, along with antioxidative and anti-inflammatory effects. The aim of this study was to evaluate the pharmacological effects of CHD on a traumatic brain injury (TBI) mouse model to explore the possibility of CHD use in patients with TBI. The TBI mouse model was induced using the controlled cortical impact method. CHD was orally administered twice a day for 5 d after TBI induction; mice were assessed for brain damage, brain edema, blood-brain barrier (BBB) damage, motor deficits, and cognitive impairment. Treatment with CHD reduced brain damage seen on histological examination and improved motor and cognitive functions. However, CHD did not reduce brain edema and BBB damage. In conclusion, CHD could be a candidate agent in the treatment of patients with TBI. Further studies are needed to assess the exact mechanisms of the effects during the acute-subacute phase and pharmacological activity during the chronic-convalescent phase of TBI.

## 1. Introduction

Traumatic brain injury (TBI) is a brain pathology induced by mechanical forces and can be divided into two pathological states, primary and secondary brain damage. The former is characterized by contusion and hemorrhage induced by mechanical forces, such as shearing, tearing, or stretching at the beginning of brain damage. The latter is characterized by delayed neuronal loss and neurological dysfunction triggered by primary brain damage. The pathophysiological mechanisms in secondary brain damage have been well documented [1, 2]. Several studies of various mechanisms in the past decades have shown no significant improvement in TBI treatment [3, 4].

Therefore, it is important to continue developing therapeutic interventions that could be helpful in attenuating the deleterious results of TBI and to search for other interventions used in the traditional medical clinics of East Asia that could be used in the treatment of TBI. In research examining traditional medicines, various studies have been conducted to examine the potential of traditional medicines as clinical interventions for TBI. To date, several herbs and prescriptions of traditional medicines, including Drynariae Rhizoma [5] and MLC901 [6], have been shown to ameliorate the effects of TBI [5–9].

Chunghyul-Dan (CHD) is a encapsulated agent made of an 80% ethanol extract of a preparation composed of Scutellariae Radix, Phellodendri Cortex, Gardeniae Fructus, and Rhei Radix et Rhizoma. It is prescribed most frequently for the prevention, treatment, or inhibition of secondary attacks in patients with stroke in Kyung Hee University Korean Medical Hospital [10, 11].

CHD has been reported to have various pharmacological effects. It has been shown to have beneficial effects on the vascular system, including hypocholesterolemic effects in hyperlipidemic rats and humans [12, 13], inhibitory effects on vascular cell adhesion molecule-1, and stimulatory effects on NO production in human endothelial cells [14]. It has also shown anti-inflammatory effects, including inhibitory effects on inducible nitric oxide synthase, prostaglandin E2 production in lipopolysaccharide-stimulated RAW264.7 macrophage cells [15], and ameliorating effects on swelling by reducing the mRNA levels of cyclooxygenase-2, interferon-gamma, and interleukin- (IL-) 4 in oxazolone-induced mouse ear dermatitis [16].

Regarding neurological diseases, it was shown to protect PC12 cells and rat primary hippocampal cells against *β*-amyloid (A*β*) oligomer 1–42 toxicity by inhibiting NO, tumor necrosis factor-*α*, and IL-1*β* production in microglial cells [17], protecting the brain damage and cognitive impairment against A*β* oligomer 1–42-induced brain damage by inhibiting the activation of astrocytes and microglia [17]. It also showed a neuroprotective effect against hypoxia-reoxygenation-induced neuroblastoma 2a cell damage via an antiapoptotic mechanism [18]. Clinical studies of CHD have shown beneficial effects in patients with stroke. Briefly, it was shown to decrease systolic blood pressure in stroke patients with stage 1 hypertension [19], improved arterial stiffness in patients with increased pulse wave velocity [20], and reduced the odds ratio of stroke recurrence by 77% compared with antiplatelet agent-managed controls [11].

Taking together the pathological mechanisms of TBI and the pharmacological reports of CHD, we proposed Chunghyul-Dan (CHD), an agent of Korean medicine, as a possible intervention for patients with TBI along with stroke. Thus, we evaluated the pharmacological effects of CHD in a TBI mouse model. A TBI mouse model induced by controlled cortical impact, which is a well-known model, was used to evaluate the effects of CHD on histological injury, blood-brain barrier (BBB) damage, brain edema, and functional deficits.

## 2. Materials and Methods

### 2.1. CHD Preparation

CHD was obtained from Kyung Hee Medical Hospital (Code: HH333, Seoul, Korea), which was the same preparation used for the treatment of patients in the hospital. The preparation and standardization of CHD are well described [11, 15, 17–19]. Briefly, each dried component herb including Scutellariae Radix, Coptidis Rhizoma, Phellodendri Cortex, Gardeniae Fructus, and Rhei Radix et Rhizoma was extracted by boiling with 80% ethanol for 2 hours and then mixed in 4 : 4 : 4 : 1 after evaporation and lyophilization, respectively.

### 2.2. Animals

All surgical procedures were approved by the Kyung Hee University Institutional Animal Care and Use Committee (KHUASP(SE)-15-102). ICR mice (DaeHan BioLink, Korea) were acclimatized for 1 week to controlled temperature conditions (22 ± 2°C), with constant humidity, and a 12 h light/dark cycle. Food and water were available ad libitum. Mice were deprived of food overnight with free access to water before surgery.

### 2.3. TBI Induction

The TBI mouse model was induced by a modified controlled cortical impact (CCI) protocol [21]. Briefly, mice (25–30 g) were anesthetized with isoflurane and placed on a stereotaxic frame. A 4 mm circular craniotomy was performed on the right hemisphere by using an electric drill (−2 mm anteroposterior and 2 mm mediolateral to the bregma). Trauma was induced with an electric impact device (Leica, USA) using a rounded impact tip (2.5 mm) at a velocity of 2 m/sec with a depth of 2 mm and a duration time of 3 msec. After impact, the surgery site was recovered by the skull and glue applied for fixation. Body temperature was monitored using a rectal thermometer and maintained at 37 ± 0.5°C during surgery by using a heating pad. Postoperatively, mice were allowed to recover in a cage. Sham-operated animals underwent craniotomy without impact.

### 2.4. Experimental Grouping and CHD Treatment

Mice were divided into six groups (sham, vehicle, 30, 100, 300 mg/kg CHD, and 45 mg/kg minocycline [22]) for the assessment of histological damage and seven groups by adding normal group to the six groups mentioned above for assessment of behavioral studies, brain edema, and BBB damage (*n* = 8). Normal group and sham-operated group were included for comparison in the behavioral studies. The extract was suspended in distilled water (DW) and daily administered orally at 3.3 mL/kg for the first 5 d after TBI induction. Minocycline was dissolved in normal saline and injected intraperitoneally. Vehicle-treated mice were given the same volume of DW.

### 2.5. Motor Function Assessment

Motor function was assessed using a beam walking test and balance beam test on 0 d (day), 3 d, and 7 d after induction.

The beam walking test was performed using a previously described method with minor modification [23]. A wooden rectangular bar (1 cm wide, 100 cm long, and 50 cm high) connected the start platform and goal box with a 10 cm opening. Mice underwent training sessions prior to TBI to learn to transverse the beam and enter the goal box. Data were obtained by measuring the mean transversal time and foot fault time in five trials.

The balance beam test was performed using a previously described method with some modification [23]. Briefly, mice were placed on the middle of a wooden rectangular bar (5 mm wide, 100 cm long, and 50 cm high) and scored as follows: mice unable to stay on the beam for 30 sec, 0 points; mice able to stay on the beam for 30 sec, 1 point; mice able to turn to the left or right side of the beam without walking, 2 points; mice able to turn left or right and walk on the beam with more than one step, 3 points; mice able to traverse the beam with more than 50% of foot slip of the affected hind limb, 4 points; mice able to transverse the beam with less than 50% of foot slip of the affected hind limb, 5 points; and mice able to traverse the beam with not more than one foot slip, 6 points.

### 2.6. Cognitive Function Assessment: Novel Object Recognition (NOR) Test

Cognitive function was measured using an NOR test at 8 d after TBI induction. The test was performed according to a previously described method with minor modifications [24]. Mice were placed in a black, wooden, no-top square box (45 × 45 cm size, 25 cm high walls) for 30 min daily for 3 d before test. On the trial day, mice were placed into the box with two old objects for 5 min and taken out for 1 hour. They were then placed back in the box with one new object and one old object. Recognition index was calculated as follows:

(1) $$\begin{array}{c} \text{Recognition index}=\frac{\left(time\;\;spent\;\;exploring\;\;the\;\;new\;\;object-time\;\;spent\;\;exploring\;\;the\;\;old\;\;object\right)}{\left(total\;\;time\;\;spent\;\;exploring\;\;both\;\;objects\right)}. \end{array}$$

### 2.7. Measurement of Brain Injury

The brains were fixed by perfusing with 4% paraformaldehyde at 8 d after induction and cut into 30 *μ*m sections with a Cryocut (Carl Zeiss, Germany) and stained with hematoxylin and eosin (H&E). The damaged area (%) was calculated compared with the intact hemisphere of each mouse using Image J software (NIH, USA).

### 2.8. Brain Water Contents Assay

Mice were sacrificed 48 hours after TBI induction and their brains were quickly removed and divided into damaged and nondamaged hemispheres. The wet weight of each hemisphere was measured on a chemical balance (Mettler HL52; Ohaus Co., NJ, USA) within 90 s of isolation. After drying the brain in an oven at 105°C for 48 hours, the dry weight was then obtained. The water content of each hemisphere was calculated as [wet weight − dry weight]/wet weight × 100.

### 2.9. Evans Blue (EB) Leakage Assay

EB leakage was analyzed 48 hours after TBI using a previously reported method [25], with some modifications. EB dye (2%, 5 ml/kg body weight; Sigma-Aldrich) was slowly injected into the tail vein 44 hours after TBI and allowed to circulate for 4 hours. Mice were then anesthetized and perfused with 15 mL 0.1 M phosphate buffer. After brain isolation, the pons and olfactory bulb were removed and the brain was immediately separated into ipsilateral and contralateral hemispheres. The weight of each hemisphere was measured; 2.5 times-concentrated (versus weight/volume) formamide (Sigma-Aldrich) was added and the hemispheres were homogenized. Each homogenized hemisphere was incubated at 60°C for 18 h and then centrifuged at 20,000*g* for 30 min. The absorption of the supernatant was measured at 610 nm with a spectrophotometer. A standard curve of EB in blank formamide was used to convert absorbency into concentration of EB dye. Data are presented as *μ*g of EB dye per gram tissue [26].

### 2.10. Statistical Analysis

All results are presented as mean ± SEM and were compared between groups using a one-way ANOVA followed by a post hoc Dunnett test. *P* values < 0.05 were considered statistically significant.

## 3. Results

### 3.1. Protective Effects of CHD in TBI Mouse Model

**Figure 1:**
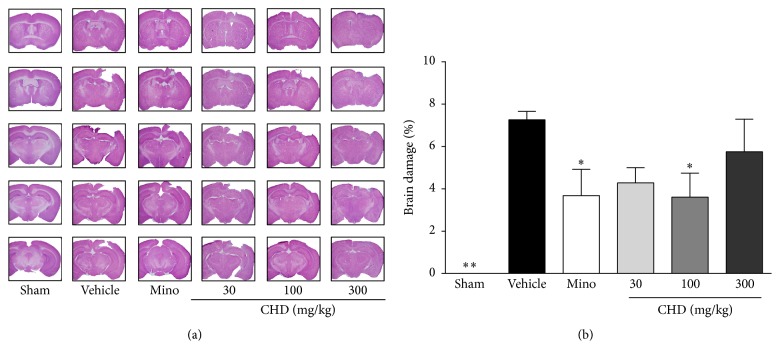
Protective effect of Chunghyul-Dan (CHD) treatment on brain damage in TBI mice model. Brain photos are the representative H&E-stained brain slices (a) and the graph shows brain damage (%) of each group (b). Values represent means ± SEM, *n* = 8. Mino is minocycline 45 mg/kg treated group. *∗* represents statistical difference from vehicle-treated group (^*∗*^*P* < 0.05, ^*∗∗*^*P* < 0.01).

The H&E-stained brain sections of the vehicle-treated group showed damage mainly in the parietal cortex that was closely related to motor function. The external capsule and corpus callosum were also damaged. Minocycline and CHD-treated groups showed less damage in those areas (Figure 1(a)). The damage in the vehicle-treated group involved 7.3% of hemisphere, while the minocycline-treated group had damage to 3.7% of the hemisphere (*P* < 0.05, Figure 1(b)). The CHD 30 and 100 mg/kg treated groups demonstrated damage in 4.3% and 3.6% of the hemisphere, respectively (*P* < 0.05, Figure 1(b)). However, the group treated with 300 mg/kg did not show any significant difference from the vehicle-treated group (5.7% of hemisphere damaged, Figure 1(b)).

### 3.2. The Effects of CHD on Brain Water Contents (BWC)

**Figure 2:**
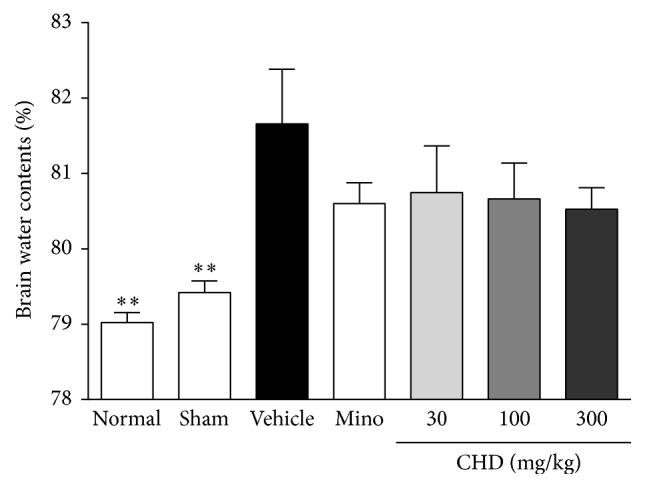
The effect of Chunghyul-Dan (CHD) on brain water content of damaged hemisphere in TBI mouse model. Values represent the mean ± SEM (*N* = 8). Mino is minocycline 45 mg/kg treated group. *∗∗* represents statistical difference from vehicle-treated group (^*∗∗*^*P* < 0.01).

The BWC of the vehicle-treated group 48 hours after TBI in the damaged hemisphere was 81.6%, while normal and sham group showed 79.0% and 79.4%, respectively. The minocycline and CHD-treated groups showed a tendency of reduced BWC compared with the vehicle-treated group. Although CHD showed a dose-dependent effect on BWC, it did not have any significant effect (80.4%, 80.6%, and 80.5% BWC in the 30, 100, and 300 mg/kg treated groups, resp.). Minocycline treatment also did not lead to a significant reduction in BWC (Figure 2).

### 3.3. The Effects of CHD on EB Leakage

**Figure 3:**
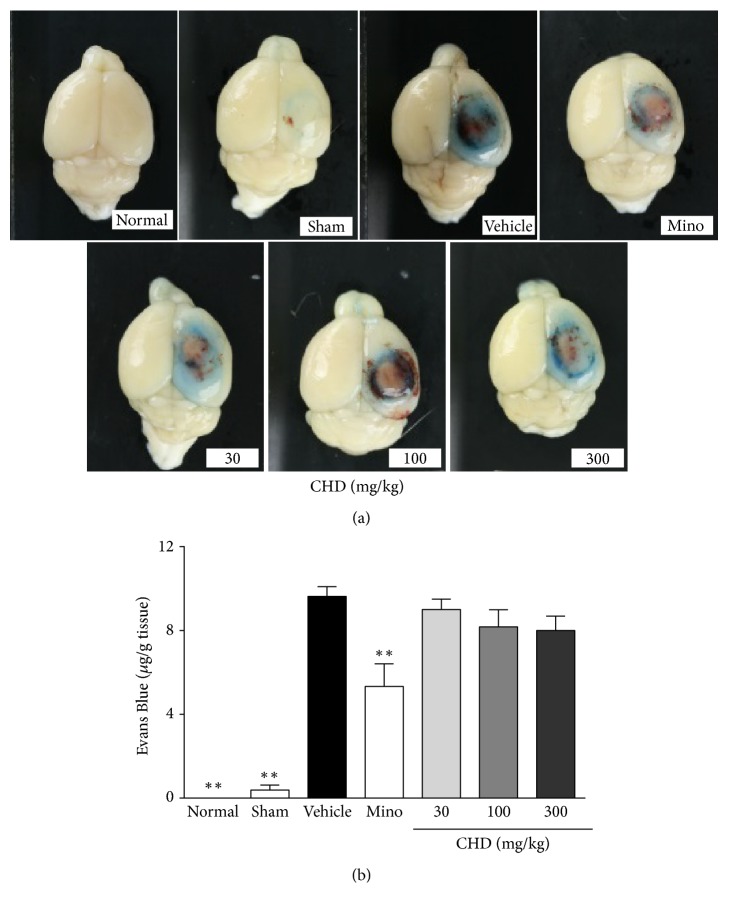
The effect of Chunghyul-Dan (CHD) on Evans Blue leakage (*μ*g/g tissue) in TBI mouse model. Photos are the representative brain of each group after Evans Blue injection (a). The graph shows the Evans Blue contents of each group (*μ*g/g tissue) after quantification (b). Values represent means ± SEM, *n* = 8. Mino is minocycline 45 mg/kg treated group. *∗∗* represents statistical difference from vehicle-treated group (^*∗∗*^*P* < 0.01).

The vehicle-treated group had increased EB leakage compared with the sham group (9.63 versus 0.38 *μ*g/g tissue) and normal group showing no EB leakage. Minocycline treatment led to reduced EB leakage compared with that of the vehicle-treated group (5.33 *μ*g/g tissue, *P* < 0.01). CHD showed a dose-dependent effect on EB leakage; however, it did not reach significance (9, 8.16, and 8 *μ*g/g tissue in the 30, 100, and 300 mg/kg treated groups, resp., Figure 3).

### 3.4. Effects of CHD Treatment on Beam Walking and Balance Beam Tests

**Figure 4:**
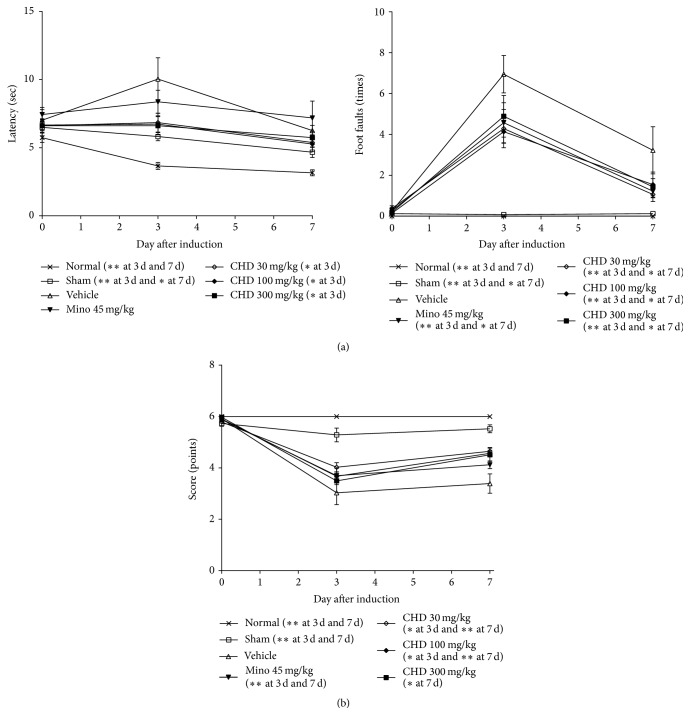
The effects of Chunghyul-Dan (CHD) on motor functional deficits in TBI mouse model using beam walking test and balance beam test. Graphs show the value at each time point (0 d (before) and 3 d and 7 d after TBI). (a) is latency (sec) and foot faults (times) of beam walking test and (b) is the score (points) of balance beam test. Values represent means ± SEM, *n* = 8. Mino is minocycline. *∗* represents statistical difference from vehicle-treated group at each time point (^*∗*^*P* < 0.05, ^*∗∗*^*P* < 0.01).

In the beam walking test, the vehicle-treated group demonstrated increased latency and number of foot faults 3 d after TBI induction. These recovered to the normal range within 7 d. The sham and normal groups did not show any functional deficits. The minocycline-treated group had a change in the number of foot faults at 3 d (*P* < 0.01) and 7 d (*P* < 0.05) after TBI but not latency. All CHD-treated groups demonstrated decreased latency (3 d, *P* < 0.05) and number of foot faults at 3 d (*P* < 0.01) and 7 d (*P* < 0.05) compared with the vehicle-treated group (Figure 4(a)).

In the balance beam test, the vehicle-treated group had lower scores at 3 and 7 d after TBI compared with the sham and normal groups that demonstrated no change in test scores before and after TBI (*P* < 0.01). While the minocycline-treated group had higher scores than the vehicle-treated group at both 3 and 7 d after TBI (*P* < 0.01, Figure 4(b)), the CHD-treated groups also showed significant improvement compared with the vehicle-treated group (^*∗*^*P* < 0.05, ^*∗∗*^*P* < 0.01, Figure 4(b)).

### 3.5. Effects of CHD in the NOR Test

**Figure 5:**
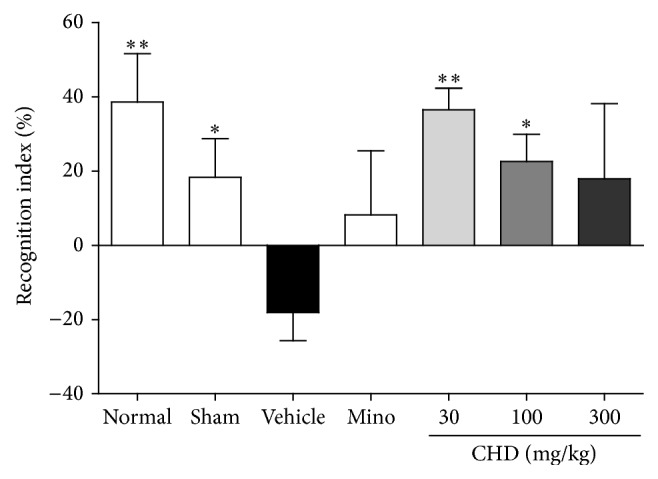
The effects of Chunghyul-Dan (CHD) on cognitive functional deficits in TBI mouse model using novel object recognition test. Graph shows the recognition index (%) of each group. Values represent means ± SEM, *n* = 8. Mino is minocycline-treated group. *∗* represents statistical difference from vehicle-treated group (^*∗*^*P* < 0.05, ^*∗∗*^*P* < 0.01).

The vehicle-treated group showed a reduced recognition index (%) compared with the sham and normal groups (*P* < 0.01). The CHD-treated groups (30 and 100 mg/kg) had higher recognition indices than the vehicle-treated group (*P* < 0.05). However, the CHD 300 mg/kg treated and minocycline-treated groups did not show any significant differences from the vehicle-treated group (Figure 5).

## 4. Discussion

In the current study, CHD showed protective effects against brain injury and motor and cognitive functional deficits in a TBI mouse model.

One of the most significant findings of the current study was that CHD ameliorated the effects of brain injury without reducing brain edema or BBB damage. The pathophysiology of TBI is well documented. The brain damage begins with a short-term primary phase caused by direct mechanical force-induced tissue distortion and destruction. It may be worsened by secondary phase brain injury shortly after the initial injury. The secondary phase is characterized by further ischemic or hemorrhagic pathological cascades including inflammation, oxidative stress, and apoptosis [26]. In the current study, the effects of CHD on brain injury might be supported by antioxidative [27, 28], antiapoptotic [18], anti-inflammatory [14, 15, 17, 29], and neuroprotective effects [17, 18, 27], an inhibitory effect of adhesion molecules including vascular cell adhesion molecules [14], and protective effects against stroke [11, 18]. Furthermore, the component herbs also have well-documented activities related to the mechanisms of brain damage [30–37].

However, CHD did not inhibit brain edema or BBB damage. The current results indicated that the protective effect of CHD against brain damage might not be correlated with these phenomena in the acute phase but partly with the subsequent cascades including apoptosis or neuroinflammation.

A second finding of the current study is that CHD improved motor functions as evaluated by the beam walking and balance beam tests. These tests are well known and have been widely used in studies of various models of brain injury and motor functional deficits to evaluate the sensory motor function, motor coordination, and balance [38–40]. In the current study, the functional tests were performed at 3 and 7 d after TBI induction. Motor function is known to be obviously affected from 1 d to 3–5 d after TBI induction and recovered by 5 d [41–43]. The current results lead to two potential conclusions. The first is that CHD might have beneficial effects for motor function after TBI that could be utilized in the chronic rehabilitation phase or in disease models associated with motor functional deficits. The second is that these effects might result from protective effects against damage to the cortex, which is the main processing center for limb movement. It could be used as further evidence for the effects of CHD against brain damage.

In addition to motor function, CHD also led to improved cognitive function as evaluated by the NOR test, which is a simple and representative tool to evaluate cognitive function in rodent models [44]. It is designed based on the natural preference of animals for a new object and helps evaluate cognitive and memory function with two different objects [44, 45]. The cognitive dysfunction in TBI is known to be obvious from 7-8 d after injury [46] and influenced by both hippocampal and cortical lesions, specifically lesions in the perirhinal cortex and medial temporal lobe [47]. The current result might be due to the reduction of damage to the parietal cortex and hippocampus. The current result could be also supported by previous studies of CHD and its component herbs that demonstrated memory enhancing effects [48–52]. We could not identify the cause of the ineffectiveness of minocycline; it may be due to a statistical issue.

The positive control condition used in the current study, minocycline, has been reported to have protective effects against various brain injuries including TBI [47, 53, 54]. The dose of 45 mg/kg was chosen based on a previous report [47]. The dose of CHD in the current study was chosen with respect to the doses used clinically, which is typically 15 mg/kg a day (300 mg capsule, three times a day in a 60 kg human). The doses used in the current study, 30, 100, and 300 mg/kg (twice a day), correspond to 4, 13.3, and 40 times the typical human dosage. In general, the dose in mice could be considered to be approximately 12.3 times higher than that in a human according to the formula for dose translation [55]. Thus, 30–100 mg/kg might be the optimal dosage to use in murine studies.

## 5. Conclusion

CHD treatment could ameliorate brain injury and injury-related motor and cognitive functional deficits in a TBI mouse model at doses of 30 and 100 mg/kg. CHD could be a candidate agent of Korean medicine for patients with TBI, in addition to its current use in patients with stroke. Further studies are needed to assess the exact mechanisms of the effects in the acute-subacute phase and pharmacological activity during the chronic-convalescent phase of TBI.
